# Dried Human Amniotic Membrane Does Not Alleviate Inflammation and Fibrosis in Experimental Strabismus Surgery

**DOI:** 10.1155/2013/369126

**Published:** 2013-06-23

**Authors:** Bo Young Chun, Hong Kyun Kim, Jae Pil Shin

**Affiliations:** Department of Ophthalmology, School of Medicine, Kyungpook National University, Daegu, Republic of Korea

## Abstract

*Purpose*. The purpose of this study was to evaluate the efficacy of dried human amniotic membrane (AM) in reducing the postoperative inflammatory response and scarring after strabismus surgery. *Methods*. The inflammatory response at the extraocular muscle reattachment site was analyzed after superior rectus (SR) resection in 12 rabbits. Dried human AM (Ambiodry2) was applied between the resected SR muscle plane and Tenon's capsule of the left eyes of rabbits. As a control, the right eyes of rabbits underwent SR resection only. The surgeon randomly ordered which eye gets operated first during the experiment. Two weeks later, enucleation was performed. Six sagittal sections were made for each eye at the insertion of the SR muscle. The grade of postoperative inflammation and the presence of fibrosis were evaluated in histological examinations. *Results*. There was no statistically significant difference in the intensity of inflammation and fibrous proliferation between the eyes treated with dried human AM after SR resection and those treated with SR resection only. *Conclusions*. The use of dried human AM was not effective in controlling the postoperative inflammation and scarring in rabbit eyes after extraocular muscle surgery. However, this may be due to the devitalized dry preparation of human AM (Ambiodry2), which may have lost the expected anti-inflammatory and anti-scarring properties, and further studies on humans may be necessary.

## 1. Introduction

Postoperative adhesion is one of the major complications of strabismus surgery that can cause motility problems that affect the surgical outcomes [1, 2]. Due to the fact that the suppression of inflammation is a key element in the prevention of further fibrovascular proliferation and scar formation in the conjunctiva [3], various approaches have been used to reduce postoperative inflammation and scarring following strabismus surgery. Among these are mechanical barrier devices that isolate the muscle from the sclera and the Tenon's capsule, steroids, antiproliferative agents and viscoelastic substances [1–9]. However, none of these techniques has been widely accepted because of associated complications and inconsistent results.

The use of the amniotic membrane (AM) in other applications of ophthalmology such as ocular surface reconstruction, filtration surgery, and the treatment of pterygium has increased [3, 10–12]. The AM can promote epithelialization of the cornea and the conjunctiva and reduce inflammation, scarring, and neovascularization in anterior segment surgeries [13, 14]. AM exhibits several characteristics that might be of benefit in strabismus surgery, that is, good integration with surrounding tissue, a low-healing response, suppression of transforming growth factor-beta (TGF-*β*) activity, and poor immunogenicity [15].

Although it has been reported that cryopreserved AM reduced adhesions after strabismus surgery [14], there is no report describing the anti-inflammatory and anti-scarring properties of dried human AM after strabismus surgery. In contrast to cryopreserved human AM, dried human AM is low-electron-beam sterilized and preserved using low heat and air vacuum [16, 17].

The purpose of the current study was to evaluate the efficacy of dried human AM in reducing the postoperative inflammatory response and scarring after strabismus surgery in rabbits.

## 2. Materials and Methods

### 2.1. Animals

A prospective, controlled experiment was performed. Twelve New Zealand white rabbits weighing 2-3 kg were used in this study. The procedures followed the recommendations of the Association for Research in Vision and Ophthalmology (ARVO) Statement for the Use of Animals in Ophthalmic and Vision Research. 

### 2.2. Surgery

Both eyes of all rabbits underwent strabismus surgery consisting of a 4 mm resection of the superior rectus (SR) muscle in a standard manner. All surgeries were performed by the same surgeon (BYC). The animals were anesthetized with an intramuscular injection containing a mixture of ketamine hydrochloride (Ketalar, Parke Davis, 20 mg/mL) at 25 mg/kg and an aqueous solution of 2% xylazine (Rompum, Bayer, 7 mg/mL) at 5 mg/kg. The anesthesia level was monitored by blink reflex and toe withdrawal reflex. A povidone-iodine solution was then applied to the eyelids for preoperative antisepsis.

The SR muscle was exposed through a fornix-based incision. The SR muscle was isolated on a muscle hook, and the intermuscular membranes and superior oblique muscle were removed by careful dissection with Wescott scissors. A double-armed 6-0 polyglactin suture (Vicryl) was placed on the SR muscle 1 mm behind the resection point, and a 4 mm resection of the SR muscle was performed. To intensify the inflammatory response, cautery of the underlying scleral bed (1 × 0.5 cm^2^, 10 times) was performed before reattachment of the resected SR muscle [18]. The reason of the intensification of the inflammation is that we do not know the exact degree of the inflammatory response of strabismus surgery performed in rabbits. De Carvalho et al. [7] reported that there was only a minimal inflammatory response after a simple strabismus surgery in rabbits. Therefore, we intentionally enhanced the inflammation to verify the anti-inflammatory effect of the AM easily.

In the left eyes, a half sheet of dried human AM with an intact monolayer of epithelium (Ambiodry2; Okto Ophtho, Costa Mesa, CA, USA) (1.5 × 1.5 cm) was inserted between the resected SR muscle plane and the Tenon's capsule. The less-reflective surface of the AM (stromal side) was placed toward the muscle plane to handle the AM easily during surgery because the stromal side of the AM is relatively rough and adhesive, while the epithelial side of the AM is shiny and smooth. In addition, the fibrotic proliferation was expected to come from the subconjunctival fibroblasts [1, 9]. 

The conjunctiva was then repositioned with caution and closed with two 6-0 polyglactin sutures in all eyes. The contralateral eyes of the rabbits underwent SR resection only. During this experiment, the surgeon randomly ordered which eye gets operated first. Postoperatively, an antibiotic ointment (Terramycin) was used. 

### 2.3. Enucleation and Histopathological Examination

Two weeks after the surgery, all rabbits were reanesthetized. The eyes were carefully enucleated without causing damage to the scleromuscular junction. An incision was made at the superior conjunctiva, 12 mm posterior to the limbus, and the SR muscle was cut at that location. Then, the incision was extended to the 3 and 9 o'clock positions, and the remaining muscles and optic nerve were cut. Finally, the rabbits were euthanized.

For the histopathological examination, the enucleated eyes were fixed in 10% buffered formaldehyde and embedded in paraffin. Six consecutive sagittal sections for each eye were made perpendicularly to the line of the postsurgical insertion of the SR muscle. The tissues were processed for histological examinations through staining with hematoxylin eosin (HE) and Masson's trichrome (MT) to evaluate the severity of inflammation and the presence of fibrosis. These staining methods identify the muscular cell nuclei as blue-black, cytoplasm as red, staple fibers as red, and collagen as blue [7].

**Figure 1:**
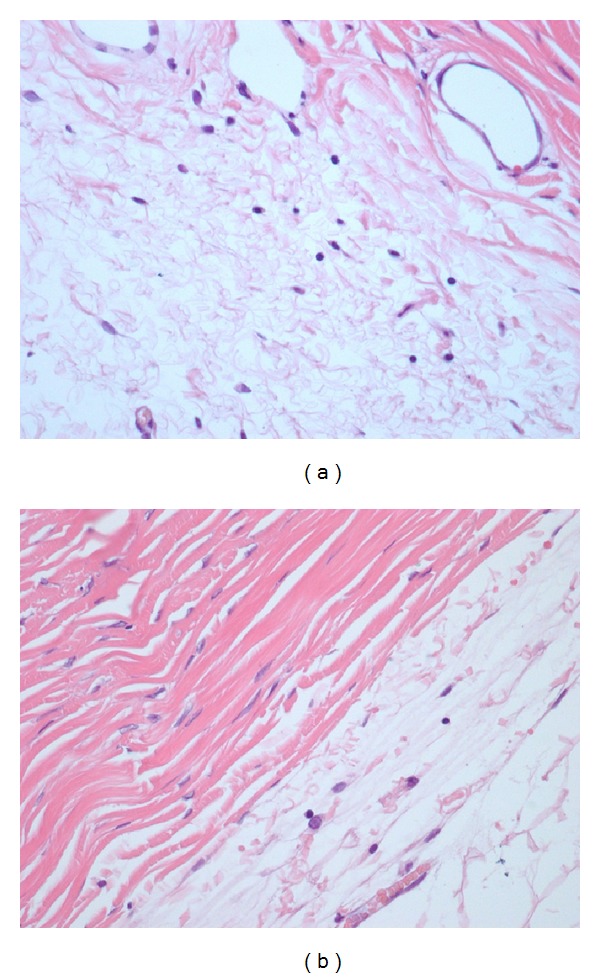
Grade 1 inflammation (presence of lymphocytes) in a control eye (a) and an AM eye (b) with H&E stain and 400x magnification.

**Figure 2:**
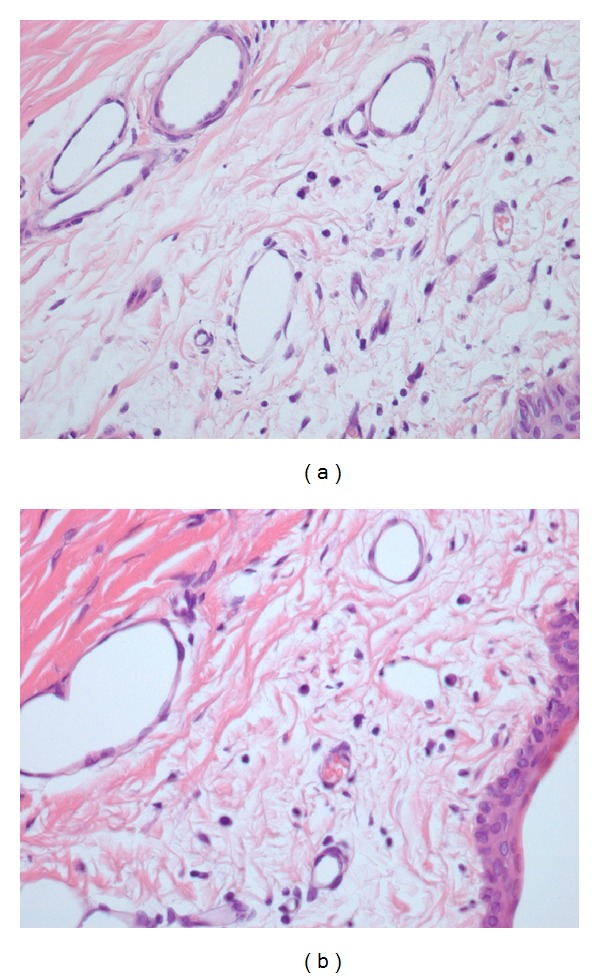
Grade 2 inflammation (presence of lymphocytes, plasmocytes, and macrophages) in a control eye (a) and an AM eye (b) with H&E stain and 400x magnification.

**Figure 3:**
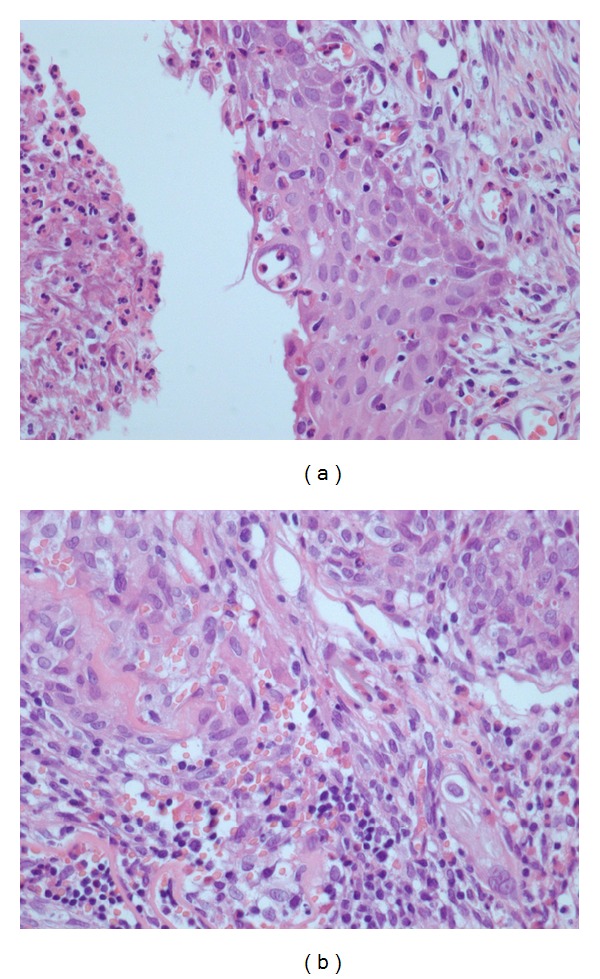
Grade 3 inflammation (presence of lymphocytes, plasmocytes, macrophages, and neutrophils) in a control eye (a) and an AM eye (b) with H&E stain and 400x magnification.

Inflammation was graded through semi-quantitative analysis under light microscopy by a pathologist who was blinded to the treatment groups (Figures 1, 2, and 3) [7, 19].

The following grading system was used:

(0): inflammatory infiltrate absent,
(1): mild inflammatory infiltrate (presence of lymphocytes),
(2): moderate inflammatory infiltrate (presence of lymphocytes, plasmocytes, and scattered macrophages),
(3): intense inflammatory infiltrate (presence of lymphocytes, plasmocytes, macrophages and neutrophils).

After grading inflammatory scores of six sections per each eye, the number of whole sections with each grade (absent, mild, moderate, or intense) was determined for the comparison of grades of inflammation.

Fibrosis was defined as collagen deposition between the SR muscle and the Tenon's capsule, which was stained blue with MT. The ocular tissue was examined with 100x and 400x magnification (Elipse E 600, Nikon, Japan). For comparison of fibrosis, the number of whole sections with showing fibrosis was determined.

### 2.4. Statistical Analysis

Fisher's exact test was used to determine the relationship between the severity of inflammation (absent, mild, moderate, or intense) and the application of dried human AM (dried human AM or control). This test was also used to determine the relationship between the presence of fibrosis (absent or present) and the application of dried human AM (dried human AM or control). Statistical analyses were performed with SPSS version 12.0 (SPSS, Inc., Chicago, IL, USA) software. In all tests, the level of statistical significance was set at 5% (*P* < 0.05).

## 3. Results 

Table 1 shows the severity of postoperative inflammation after strabismus surgery in the dried human AM eyes and the control eyes. The degree of inflammation was graded in 6 consecutive sagittal sections in each eye. There was no statistically significant difference in the grade of inflammation between the control eyes and the AM eyes (*P* = 0.177, Fisher's exact test).

Table 2 shows the extent of postoperative fibrosis in each group. There was no statistically significant difference in the number of sections that showed fibrosis with Masson's trichrome stain between the control eyes and the AM eyes. (*P* = 0.428, Fisher's exact test). The statistical analysis indicated that the intensity of the inflammation and the presence of fibrosis are not affected by the application of dried human AM (*P* > 0.05).

**Figure 4:**
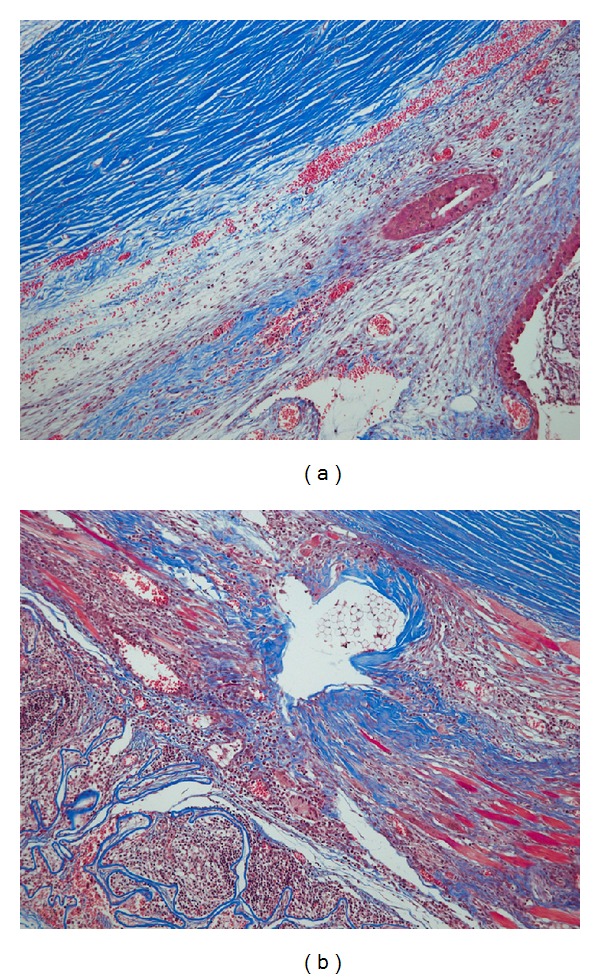
Fibrosis in a control eye (a) and an AM eye (b) with Masson's trichrome stain and 100x magnification.

The AM remained intact on histologic examination after 14 days but were associated with marked infiltration of inflammation cells with surrounding fibrosis around the AM (Figure 4). 

## 4. Discussion

The amniotic membrane (AM) is the innermost layer of the placenta and consists of a thick basement membrane and a subjacent avascular stromal matrix [3, 13, 20, 21]. The AM reduces the inflammation in a variety of ocular surface disorders through several mechanisms. One of the most important clinical characteristics of AM is that there is no rejection phenomenon after AM transplantation due to the lack of major histocompatibility antigen (MHA) expression [13]. In addition, the AM reduces the mixed lymphocyte reaction, delayed hypersensitivity reaction, and neovascularization [13, 20], and it suppresses the interleukin-1 (IL-1)-mediated inflammation cascade [21]. In vivo rabbit studies demonstrated that the AM trapped leukocytes and rendered them into a state of rapid apoptosis [22, 23]. Therefore, we presumed that AM may be a useful adjunct to decrease postoperative inflammation and subsequent scarring not only in ocular surface disorders but also in strabismus surgery.

This study investigated the effects of dried human AM on the inflammatory response and fibrosis caused by strabismus surgery in rabbits. In this experiment, AM-treated eyes have failed to demonstrate less inflammation than control eyes, and moderate inflammatory processes were prevalent in both conditions. Furthermore, there was no statistically significant difference in the number of fibrosis-containing slide sections between AM-treated eyes and control eyes. The suppression of inflammation is a key element in the prevention of further fibrovascular proliferation and scar formation in the conjunctiva [3]. Considering that fibrosis is an important sequela of granulomatous inflammation [24], we could not regard dried human AM as an effective biological barrier against postoperative fibrosis after strabismus surgery in rabbits, although a detailed measurement of fibrosis was not obtained in this experiment.

These findings are in contrast to earlier studies reporting that AM transplantation reduced postoperative adhesions in strabismus surgery. Sheha et al. [14] reported that wrapping the extraocular muscle with a sheet of cryopreserved AM reduced adhesions after strabismus surgery; however, this study was limited to only one case. Kersey and Vivian [25] reported that simultaneous application of mitomycin C and AM transplantation reduced postoperative fibrosis in two patients with complicated strabismus surgery. However, it is difficult to conclude that the antifibrosis effect observed in this report was due to the AM transplantation only. The most important difference to note is that these were human-human allograft transplantations, whereas our study used a rabbit-human xenograft model.

We speculate that the AM failed to decrease the inflammation in this experiment for the following reasons. First, there may be a possibility of xenograft rejection phenomenon. We were interested in the unexpected nonsignificant trend of increased infiltration of inflammatory cells and fibrosis especially around the applied AM (Figure 4). Barton et al. [26] reported significant granulomatous inflammation with giant cell formation after 14 days that was classified as a xenograft reaction, which was a time point similar to our study. These findings may be a sign of undesirable xenograft rejection, although the dried human AM used in this experiment does not carry any live cells. Second, the stromal matrix of AM traps leukocytes and renders them into a state of rapid apoptosis [22, 23], and this phenomenon occurs in corneal limbal epithelial cells. In this experiment, the AM apparently did not block leukocyte infiltration in a different environment, that is, the space between the extraocular muscle and the Tenon's capsule in strabismus surgery. Third, dried human AM (Ambiodry2) is manufactured by a dehydration process using low heat and air vacuum, and it lacks viable cells [17]. Devitalized dry preparation of human AM (Ambiodry2) may have lost the expected anti-inflammatory and anti-scarring properties that are noted in cryopreserved AM. In addition, study of Thomasen et al. [27] reported that cryopreserved AM demonstrated a higher outgrowth rate of cultured limbal epithelium, release of intact soluble wound-healing modulating factors, and better preservation of basement membrane components than air-dried AM, which supports our hypothesis. The differences between cryopreserved AM and dried AM seem to be caused by the different preservation and sterilization processes [27].

This study has some important limitations that mostly stem from its small number of animals and the relatively short duration before enucleation. The authors set the time of enucleation at 2 weeks postoperatively because the completion of tissue repair is usually occurs 10 to 14 days after strabismus surgery [28]. However, a longer duration may be necessary to evaluate the degree of fibrosis after the complete absorption of the AM. In addition, we evaluated the presence of fibrosis only between the extraocular muscle plane and the Tenon's capsule and not between the sclera and the muscle plane. This was due to the results of a previous study, which reported a lack of fibrosis between the muscle plane and the sclera after strabismus surgery [9]. It is also not clear whether the activity of TGF-*β* has decreased by the application of the AM in this experiment model.

This is the first experimental study to evaluate the effect of dried human AM as a biological barrier against postoperative fibrosis via downregulation of inflammation in strabismus surgery. 

In conclusion, there was no statistically significant difference in the intensity of inflammation and fibrous proliferation between rabbit eyes treated with dried human AM after SR resection and rabbit eyes receiving SR resection only. Further studies with immunosuppressive agents in the same model to inhibit potential xenograft-drived inflammation, however, are needed to further confirm the role of the dried human AM as a biological barrier in strabismus surgery.

## Figures and Tables

**Table 1:** The number of sagittal sections (6 sagittal sections per rabbit eye) with each grade of inflammation after strabismus surgery according to the treatment (dried human amniotic membrane (AM) or control).

Grades of inflammation	Absent	Mild	Moderate	Intense	Total
	*N* (%)	*N* (%)	*N* (%)	*N* (%)	*N* (%)
Control	0 (0%)	4 (5.6%)	51 (70.8%)	17 (23.6%)	72 (100%)
Dried human AM application	2 (2.8%)	6 (8.3%)	40 (55.6%)	24 (33.3%)	72 (100%)

(*P* = 0.177, Fisher's exact test).

**Table 2:** The number of sagittal sections with and without fibrosis among 24 eyes of rabbits (6 sagittal sections per eye) after strabismus surgery according to the treatment (eyes with dried human AM application versus control eyes).

	Presence of fibrosis	No fibrosis	Total
	*N* (%)	*N* (%)	*N* (%)
Control	53 (73.6%)	19 (26.4%)	72 (100%)
Dried human AM application	58 (80.6%)	14 (19.4%)	72 (100%)

(*P* = 0.428, Fisher's exact test).
